# GC-MS Analysis and Study of the Antimicrobial Activity of *Citrus paradisi*, *Citrus aurantifolia*, and *Citrus sinensis* Peel Essential Oils as Hand Sanitizer

**DOI:** 10.1155/2024/4957712

**Published:** 2024-01-02

**Authors:** Isra Osman Mohammed, Ayat Ahmed Alrasheid, Saad Mohammed Hussein Ayoub

**Affiliations:** ^1^Pharmaceutical Analysis and Quality Control Program, Graduate College, University of Medical Sciences and Technology, Khartoum, Sudan; ^2^Pharmacognosy Department, Faculty of Pharmacy, University of Medical Sciences and Technology, P.O. Box 12810, Khartoum, Sudan

## Abstract

In the setting of healthcare, the use of hand sanitizers and antiseptics for hand hygiene is of paramount importance to avoid transfer of pathogenic microorganism through hand and skin contact. There is an increasing interest in the incorporation of essential oils in hand sanitizer's formula to avoid the adverse effect of conventional hand sanitizers on health. This study aimed to detect the chemical constituents of citrus peel essential oils and study their antimicrobial activity compared with commercial hand sanitizers. The qualitative and quantitative analysis of the hydrodistillated essential oils of peels of grapefruit (*Citrus paradisi*), lime (*Citrus aurantifolia*), and orange (*Citrus sinensis*) were carried out using gas chromatography mass spectroscopy. The disc diffusion method was used to screen the antibacterial activity of the essential oils against *Escherichia coli*, *Pseudomonas aeruginosa*, *Bacillus subtilis*, *Staphylococcus aureus*, and *Candida albicans* compared with a 78% alcohol-based commercial hand sanitizer. The antimicrobial testing results were statistically analyzed. The highest yield percentage of the obtained essential oils was 1.09% obtained by orange oil. The GC-MS analysis indicated that monoterpene and sesquiterpene hydrocarbons occupied the largest portion of the chemical composition of the three essential oils with D-limonene as the most predominant component. All essential oils showed activity against all tested organisms. Lime essential oil showed comparable antimicrobial activity relative to the commercial 78% alcohol hand sanitizer. In conclusion, essential oils obtained from citrus fruit peel represent a rich source of compounds possessing antimicrobial properties and could be an alternative to synthetic antimicrobial agents.

## 1. Introduction

In the setting of healthcare, the use of hand sanitizers and antiseptics for hand hygiene is of paramount importance to avoid transfer of pathogenic microorganism through hands and skin contact [1]. Antiseptics are chemical compounds with antimicrobial activity that can be used on skin and mucosal surfaces due to their low toxicity [2]. Most antiseptic agents can damage the skin, leading to a change in microbial flora; hence, an increased shedding of the original protective bacterial flora of the hand leads to an increased risk of transmission of pathogenic microorganisms [3]. Furthermore, the use of an alcohol-based hand rub is the preferred method of hand hygiene according to recently revised hand hygiene guidelines compared to alcohol-free hand sanitizers. However, given the health effects of alcohol ingestion, it can be assumed that alcohol absorption through inhalation and to a lesser extent via dermal contact might induce negative long-term health effects. Some healthcare workers have complained of an unpleasant smell associated with the use of alcohol rubs [4]. Another concern related to alcohol-based hand sanitizers is fire hazard [5]. Recently, interest in essential oils has been revived as a natural alternative to conventional treatments and therapeutic practices [6]; they have been characterized for their carcinogenic effect, acute toxicity, and environmental hazard potential [7].

Essential oils are natural aromatic compounds isolated from plants. Oil is “essential” in the sense that it contains the “essence of” the plant's fragrance. These complex mixtures have been used medicinally throughout history for a wide range of purposes. One area of study related to the bioactivity of essential oil involves the use to combat microbes. Many research studies have focused on the exploration of the features of essential oil to synergize or even to compete with antiseptic agents [4, 6, 8, 9].

Citrus plants belong to the family *Rutaceae*, comprising about 17 species found throughout the tropical, subtropical, and temperate regions. The genus *Citrus* includes different important fruits such as orange (*Citrus sinensis*), mandarins (*Citrus reticulata*), limes (*Citrus aurantifolia*), lemons (*Citrus limon*), and grapefruits (*Citrus paradisi*). Although, there are many groups of plants that are essential in phytochemistry, citrus plantation has been considered the most valuable for industrial and commercial agricultural practices in the world [10]. Several studies have demonstrated the potential of terpenes in citrus essential oils to inhibit the growth and activity of various microorganisms, including bacteria, fungi, and viruses. These bioactive compounds possess unique chemical properties that allow them to disrupt microbial cell membranes, inhibit enzyme activity, and interfere with essential biological processes within the microorganisms. Moreover, terpenes have shown broad-spectrum antimicrobial effects, making them promising candidates for combating drug-resistant pathogens [11]. However, citrus essential oils are generally nontoxic, nonmutagenic, and noncarcinogenic. They are not hazardous in pregnancy and do not alter the maternal reproductive outcome. Sweet orange, bitter orange, neroli, petitgrain, lime (both distilled and expressed), bergamot, and grapefruit oils are generally recognized as safe (GRAS). However, there is a possible skin sensitization issue if old or oxidized oil is used [12]. This study explored the potential of citrus essential oils for antimicrobial activity to be used as hand sanitizers of natural origin to avoid the abovementioned problems associated with the use of alcohol-based hand sanitizers, based on previous research that investigated the safety of citrus essential oils on skin and prompted by the economic importance of citrus essential oils as byproducts of the abundant waste of the citrus fruit processing industry.

## 2. Materials and Methods

### 2.1. Materials

#### 2.1.1. Plant Material

Fruits of three citrus species, namely *Citrus paradisi* (grapefruit), *Citrus aurantifolia* (lime) and *Citrus sinensis* (orange) were obtained from a local market in Khartoum. The specimens were taxonomically authenticated at the Medicinal and Aromatic Plant and Traditional Medicine Research Institute, National Center for Research, Khartoum, Sudan.

#### 2.1.2. Test Microorganisms

Antimicrobial activity of the essential oils was assessed against standard two Gram-positive bacteria: *Bacillus subtilis* NCTC 8236 and *Staphylococcus aureus* ATCC 25923, two Gram-negative bacteria: *Escherichia coli* ATCC 25922 and *Pseudomonas aeruginosa* ATCC 27853, and against standard fungal microorganism: *Candida albicans* ATCC 7596. The test microorganisms were procured from the Medicinal and Aromatic Plant and Traditional Medicine Research Institute, National Center for Research, Khartoum, Sudan.

### 2.2. Methods

#### 2.2.1. Essential Oil Extraction Method

The essential oil was extracted from fresh fruit peels using the method described in FAO manual for quality control with slight modification [13]. The peels were cleaned, cut to small slices, and extracted using a Clevenger apparatus; the obtained oils were weighed and stored in clean container. The oil yield was calculated as follows:

(1) $$\begin{array}{c} \text{Oil yield }\left(\%\right)=\frac{\text{Volume of essential oil obtained }\left(\text{ml}\right)}{\text{weight of fresh peel }\left(g\right)}*100. \end{array}$$

#### 2.2.2. Determination of Chemical Constituents Using GC-MS Analysis

The essential oil samples were analyzed using the GC-MS technique with the Shimadzu Company's model GC/MS-QP2010-Ultra, serial number 020525101565SA. A capillary column (Rtx-5 ms-30 m × 0.25 mm × 0.25 *µ*m) was used. The sample was injected in split mode, with the instrument operating in EI mode at 70 eV. Helium was used as the carrier gas at a flow rate of 1.69 ml/min. The temperature program started at 50°C with a rate of 7°C/min, reaching 180°C. Then, the rate was changed to 10°C/min, reaching a final temperature of 280°C. The injection port temperature was 300°C, the ion source temperature was 200°C, and the interface temperature was 250°C. The sample was analyzed in scan mode in the range of m/z 40–500 charges to ratio, with a total run time of 28 min. The identification of components in the sample was done by comparing their retention index and mass fragmentation patterns with those available in the National Institute of Standards and Technology (NIST) library.

#### 2.2.3. Antimicrobial Activity

*(1) Preparation of Bacterial Suspensions*. To assess the activity of the extracted essential oils, the agar disc diffusion technique was employed [14]. Firstly, one ml aliquots of a 24-hour broth culture of the test organisms were carefully distributed onto nutrient agar slopes and then incubated at 37°C for 24 hrs. The resulting bacterial growth was harvested and rinsed with 100 ml sterile normal saline, creating a suspension containing approximately 10^8^-10^9^ colony-forming units (C.F.U) per ml. This suspension was stored at 4°C until needed. To determine the average number of viable organisms per ml of the stock suspension, the surface viable counting technique was utilized. Serial dilutions of the stock suspension were prepared using sterile normal saline solution, and 0.02 ml of the appropriate dilution were transferred onto the surface of dried nutrient agar plates using a micropipette. The plates were left undisturbed for two hours at room temperature to allow the drops to dry, and then they were incubated at 37°C for 24 hrs. After incubation, the number of colonies that developed in each drop was counted. The average number of colonies per drop (0.02 ml) was multiplied by 50 and by the dilution factor to determine the viable count of the stock suspension, which was expressed as the number of colony-forming units per ml suspension. It is important to note that a fresh stock suspension was prepared each time, and all experimental conditions mentioned above were maintained constant to ensure that suspensions with very similar viable counts were obtained.

*(2) Preparation of Fungal Suspension*. The fungal cultures were maintained on Sabouraud dextrose agar, incubated at 25°C for 4 days. The fungal growth was harvested and washed with sterile normal saline and finally suspended in 100 ml of sterile normal saline. The suspensions were stored at 4°C until used.

*(3) Testing of Antibacterial Susceptibility*. The paper disc diffusion method was used to screen the antibacterial activity of essential oils using Mueller–Hinton agar (MHA). The experiment was carried out according to the National Committee for Clinical Laboratory Standards Guidelines [15]. Bacterial suspension was diluted with sterile physiological solution to 10^8^ cfu/ml (turbidity = McFarland standard 0.5). One hundred microliters of bacterial suspension were swabbed uniformly on surface of MHA, and the inocula were allowed to dry for 5 mins. Sterilized filter paper discs (Whatman No. 1, 6 mm in diameter) were placed on the surface of the MHA and soaked with 20 μl of a solution of each essential oil and the commercial hand sanitizer as a control. The inoculated plates were incubated at 37°C for 24 hr in the inverted position. The test was carried out six times for each microorganism, and the average of all readings was taken as the zone of inhibition (mm) in each case.

*(4) Antifungal Activity*. The same method as for bacteria was used, but instead of nutrient agar, Sabouraud dextrose agar was used as the inoculation medium, incubated at 25°C for two days for *Candida albicans* [15].

*(5) Positive Control (Reference) against Standard Microorganisms*. In the present work, 78% alcohol-based commercial hand sanitizer was used as positive control for the antimicrobial tests. It was tested against reference microorganisms i.e., *B. subtilis*, *S. aureus*, *E. coli*, *P. aeruginosa*, and *C. albicans*.

#### 2.2.4. Statistical Analysis

The data were statistically analyzed with analysis of variance (ANOVA) followed by *post hoc* Tukey HSD test for group-wise comparisons. A noninferiority test was carried out using t-test. The statistical analysis was performed using an online calculator available on https://www.statskingdom.com.

## 3. Results

### 3.1. Yield Percentage of Essential Oil

The yield percentage obtained for the essential oils of grapefruit, lime, and orange was calculated. The highest yield % was 1.09% obtained for orange oil, followed by lime which gave 0.7%, while a lower yield was obtained for grapefruit (0.61%). The result of hydrodistillation-extracted citrus essential oils was similar to that reported in the literature [15].

### 3.2. Chemical Composition of Citrus Essential Oils by GC-MS Analysis

The GC-MS analysis indicated that hydrocarbons in form of monoterpenes (C_10_H_16_) and sesquiterpenes (C_15_H_24_) occupied the largest portion of the chemical composition of the three essential oils, with D-limonene as the predominant component, accounting for the % area of 91, 25, and 95 in the essential oils of grapefruit, lime, and orange, respectively. Oxygenated compounds were identified, constituting of 3% in grapefruit essential oil and 36% in lime essential oil. Orange essential oil lacked components belonging to this chemical class. It was also observed that lime essential oil contained a small percentage of nitro compounds (0.08%).

### 3.3. Antimicrobial Activity Tests

#### 3.3.1. Essential Oil Zone of Inhibition against Tested Organisms

The antimicrobial effect of the citrus essential oils was assessed by measuring the zone of inhibition against the particular tested organisms (Table 1). All essential oils showed activity against all the tested organisms that ranged between 10 and 17 mm. According to the criteria adopted by Medicinal and Aromatic Plant Research Institute, National Center for Research, Khartoum, Sudan; sample is inactive when inhibition zone <9 mm, partially active (9–12 mm), active (13–18 mm), and very active (>18 mm). The highest inhibition zone was seen in lime essential oil against *P. aeruginosa*, *S. aureus*, *B. subtilis*, and *C. albicans* while the highest inhibition zone against *E. coli* was seen with orange essential oil.

#### 3.3.2. Comparison of Activity of Citrus Essential Oils to a Commercial 78% Alcohol Hand Sanitizer as Control Using ANOVA Followed by *Post Hoc* Tukey HSD

A comparative analysis was carried out to compare the activity of the citrus essential oils with that of a locally made commercial 78% alcohol hand sanitizer considered as a control. The statistical analysis was performed with one-way ANOVA followed by *post hoc* Tukey HSD for group-wise comparison and setting significance level at *p* < 0.01. The tests hypotheses were as follows:

- ANOVA hypotheses
- H0: *µ*EO_grapefruit_ = *µ*EO_lime_ = *µ*EO_orange_ = *µ*c (there is no difference between treatment groups)
- H1: at least one of the treatment groups is different (there is a difference between treatment groups)
- where the mean of the effect of the essential oil is *µ*EO and that of the control is *µ*C. The null hypothesis is rejected when the *p* value <0.01.
- *Post hoc* Tukey HSD hypotheses for a comparison between antimicrobial agent *i* and antimicrobial agent *j* (which is the control commercial hand sanitizer in this case)
- H0: *µi*-*µj* = 0
- H1: *µi*-*µj* ≠ 0

Table 2 shows that the results of lime essential oil support the null hypothesis of no difference of activity in comparison to the commercial hand sanitizer against all test microorganisms except for *E. coli.* Grapefruit essential oil also exhibited an activity comparable to that of the control against *P. aeruginosa.* Limited antimicrobial activity exhibited by lime essential oil against *E. coli* is in line with the results of a study exploring the effect of formulating an essential oil-based hand sanitizer [8].

#### 3.3.3. Noninferiority Test of Citrus Essential Oils Relative to a Commercial 78% Alcohol Hand Sanitizer as Control Using t test

For the other combinations of essential oil and organism where the alternative hypothesis of existence of difference in activity between the assessed essential oils and the control is accepted, noninferiority testing of the particular essential oil to the control was performed to refine the alternative hypothesis using *t*-test. The underlying concept of the noninferiority test is that there still might be some clinical benefit if the essential oil is worse than the control only by some acceptable margin. Another way of saying this is that if the treatment mean is actually worse than the control mean, it may be only worse by a small, acceptable value called the margin [16, 17]. In the noninferiority test, this margin was set as 10% of the mean value of the control [18] and the significance level at *p* value <0.01.

- Noninferiority test hypotheses:
- H0: *µ*EO ≤ *µ*Control-M
- H1: *µ*EO > *µ*Control-M (the beneficial response from essential oil (EO) is worse than the control by M points or less)

The null hypothesis of inferiority is rejected when the *p* value <0.01.

The results of the noninferiority test are summarized in Table 3. Since all *p* values in Table 3 are greater than 0.01, the null hypothesis of inferiority of the selected combinations of “essential oil-organism” compared to the control cannot be rejected, and the alternative hypothesis of noninferiority is excluded.

#### 3.3.4. Comparative Effects of Grapefruit, Lime, and Orange Essential Oils on Various Microorganisms

In addition to the first ANOVA conducted to compare the antimicrobial activity of the essential oils to the commercial hand sanitizer, a second one-way ANOVA, followed by *post hoc* Tukey HSD, was carried out to compare the antimicrobial activity of the essential oils to each other against each microorganism, setting the significance level at *p* < 0.05.

Results in Tables 4–8 showed that lime essential oil exhibited maximum antimicrobial activity with a statistical significant difference (*p* < 0.05). In particular, when tested against Gram-positive bacteria *S. aureus* and *B. subtilis*, as well as fungal species *C. albicans*, lime essential oil was superior to those of grapefruit and orange, which demonstrated no significant difference (*p* < 0.05) between each other (Tables 4–8).

However, the nonsignificant difference between the activities of lime and orange essential oils in the case of *E. coli* may suggest that their activities are comparable to each other (Tables 4–8). Similarly, the activity of the essential oils of grapefruit and lime, when tested against *P. aeruginosa,* may be considered comparable, as the results were not statistically significant (Table 5).

### 3.4. Citrus Essential Oil Activity Attributed to Composition

The high antimicrobial activity of lime essential oil might be attributed to its high content of oxygenated organic compounds in comparison to the essential oils of grapefruit and lime (Tables 9–11). In addition, the presence of nitrocompounds in lime essential oil might contribute to its activity. On the other hand, the similar chemical composition of grapefruit essential oil and orange essential oil, in terms of content of hydrocarbons and oxygenated compounds, renders their activity comparable against Gram-positive bacteria *S. aureus* and *B. subtilis* as well as fungal species *C. albicans.* However, it is somewhat surprising that orange essential oil against *E. coli* and grapefruit essential oil against *P. aeruginosa* had comparable activity to lime essential oil despite the orange essential oil's lack of oxygenated compounds and the grapefruit essential oil having a low content of oxygenated compounds. Assuming an appropriate sample size, a possible explanation for this might be that these microorganisms respond to specific compounds that are common between the particular essential oil and lime essential oil. For instance, by examining Table 10 and Table 11, it can be concluded that 4(10)-thujene which is common only between lime and orange essential oils, might be responsible for their comparable activity against *E. coli*. Similarly, from Table 9 and Table 10, the comparable activity of grapefruit and lime essential oils might be attributed to some of or all their common compounds: isocaryophyllene, *β*-linalool, and *γ*-elemene.

## 4. Discussion

The objective of this study was to conduct GC-MS analysis and to carry out a comparative study of the antimicrobial effect of hydrodistillated essential oils of the peels of three citrus fruits: orange (*Citrus sinensis*), lime (*Citrus aurantifolia*), and grapefruit (*Citrus paradisi*) to evaluate their efficacy as hand sanitizers against *Bacillus subtilis*, *Staphylococcus aureus*, *Escherichia coli*, *Pseudomonas aeruginosa*, and *Candida albicans*. Numerous studies have demonstrated the antimicrobial efficacy of citrus extracts against a wide range of microorganisms [19]. The findings of the present study fit with those of several previous studies that have been conducted on the antimicrobial activity of citrus peels. A study by Tao and Liu evaluated the antimicrobial activity of citrus peels against different bacteria and fungi. The researchers found that citrus peels exhibited strong antimicrobial activity against both Gram-positive and Gram-negative bacteria [19]. Another study investigated the antibacterial activity of citrus peels against food-borne pathogens. The researchers tested the peels of different citrus fruits and found that they inhibited the growth of various bacteria, including *E. coli*, *S. typhimurium*, and *S. aureus* [20]. Another study focused on the antimicrobial activity of citrus peels against oral pathogens. The researchers found that the essential oils extracted from citrus peels, such as those from lemon, exhibited strong antimicrobial activity against oral bacteria, including *S. mutans* and *P. gingivalis* [21]. In a study published by Putnik et al., researchers evaluated the antimicrobial activity of citrus peels against drug-resistant bacteria, including methicillin-resistant *S. aureus* (MRSA) and vancomycin-resistant *E. faecium* (VRE). The study found that the essential oils derived from citrus peels showed significant antimicrobial activity against these drug-resistant bacteria. These studies highlight the potential of citrus peels as natural antimicrobial agents and suggest that they could be used in the development of new antimicrobial strategies [22].

Several studies have investigated the antimicrobial efficacy of *Citrus paradisi* extract against various microorganisms. The result of this study was clear with Okunowo et al. [23], who investigated the antimicrobial activity of grapefruit peel extract against various bacteria and fungi. The results showed that the extract exhibited significant antimicrobial activity against both Gram-positive and Gram-negative bacteria, as well as several fungal strains. Additionally, Ofeimun et al. [24] explored the antimicrobial potential of *C. paradisi* peel extract against oral pathogens, demonstrating its effectiveness in inhibiting the growth and biofilm formation of these microorganisms. Research conducted by Arsène et al. [25] investigated the antimicrobial activity of grapefruit peel extract against drug-resistant strains of *S. aureus*. The study found that the extract exhibited potent antimicrobial activity, even against methicillin-resistant *S. aureus* (MRSA) strains. Furthermore, Park and Kim [26] investigated the antimicrobial activity of grapefruit seed extract against multidrug-resistant strains of *S. aureus* and *E. coli*, revealing its potential as an alternative treatment option. On the other hand, the findings of this study were totally concordant with those of several studies conducted to investigate the antimicrobial efficacy of *C. aurantifolia* extract against different types of microorganisms. A study [27] examined the antimicrobial activity of lime peel extract against pathogenic bacteria and fungi, demonstrating significant inhibitory effects. Furthermore, Ashrafur Rahman et al. [28] investigated the antimicrobial activity of lime essential oil, obtained from *C. aurantifolia*, against oral pathogens, showcasing its potential as an alternative therapeutic agent. A study by Sánchez Aldana et al. [29] reported that the lime essential oil exhibited significant antimicrobial activity against various pathogenic bacteria, including *E. coli*, *S. aureus*, and *S. typhimurium*. The present work was clear with that obtained in a study published by Torimiro et al., who investigated the antimicrobial activity of lime peel extract against drug-resistant bacteria. The researchers found that the extract exhibited significant antimicrobial activity against methicillin-resistant *S. aureus* and multidrug-resistant *P. aeruginosa*.

The high antimicrobial activity of lime essential oil might be attributed to its high content of oxygenated organic compounds in comparison to the essential oils of grapefruit and orange and to the presence of nitrocompounds that are not detected in the other essential oils [30]. The result of the present work of antimicrobial activity of *Citrus sinensis* was found to be comparable with that reported in numerous studies which investigated the antimicrobial efficacy of *C. sinensis* extract against a broad range of microorganisms. A study published by Dhiman et al. [31] found that the essential oil exhibited significant antimicrobial effects against all tested microorganisms, including *E. coli, S. aureus, C. albicans*, and *A. niger*. Recent work with *E. coli* shows that a single mutation can confer tolerance to many lethal stressors that include antimicrobials, disinfectants, and compounds used by the immune system to kill bacteria [32]. If citrus oils behave in a similar way, caution may be required to avoid selecting tolerant mutants that block killing by antimicrobial.

The antimicrobial activity of *Citrus* spp. extracts can be attributed to the presence of various bioactive compounds, including flavonoids, coumarins, and essential oils [33]. These components possess antimicrobial properties by disrupting microbial cell membranes, inhibiting key enzymes, and interfering with vital cellular processes [34].

The present work differs from some previous studies in different aspects, in terms of plant origin, methodology, types of organisms, and advanced analysis techniques. Most previous studies have focused on individual citrus species while the present work examines the antimicrobial potential of these three specific citrus species in comparison with a commercial product.

By exploring the antimicrobial properties of these citrus peel essential oils, the research may provide evidence for their potential use as a safer and more sustainable option for hand hygiene. Additionally, if proven effective, these essential oils could offer a solution in regions where access to commercial hand sanitizers is limited. Furthermore, understanding the antimicrobial activity of these citrus peel essential oils can contribute to the broader field of natural product research and potentially inspire further investigations into their applications in various industries, such as pharmaceuticals, cosmetics, and food preservation.

The research findings support the potential to promote the use of natural resources and advance the development of alternative hand sanitizers with potential benefits for public health and environmental sustainability.

## 5. Conclusion

Citrus extracts exhibit significant antimicrobial activity against a diverse array of microorganisms. Their potential applications in medicine, natural products, and food preservation make them a valuable natural resource. In addition, the antimicrobial properties of citrus extracts hold great potential as an ecofriendly and sustainable approach to combating microbial infections and offer promising prospects for the development of novel antimicrobial agents. Further research is necessary to unravel the precise mechanisms of action, optimize extraction methods, and evaluate its safety and efficacy in different applications and to develop new formula as hand sanitizer.

## Figures and Tables

**Table 1:** Zone of inhibition (mm) of the citrus essential oils and the control against test organisms.

Test organism		MDIZ^*∗*^			
		Grapefruit essential oil	Lime essential oil	Orange essential oil	Commercial 78% alcohol hand sanitizer
Gram-negative bacteria	*E. coli*	10 ± 0.41	12 ± 0.98	13 ± 1.41	18 ± 1.22
	*P. aeruginosa*	14 ± 1.47	15 ± 1.79	12 ± 1.10	17 ± 2.32

Gram-positive bacteria	*S. aureus*	10 ± 0.89	15 ± 3.08	10 ± 0.41	15 ± 1.17
	*B. subtilis*	13 ± 1.21	16 ± 0.82	13 ± 1.03	17 ± 1.75

Fungi	*C. albicans*	14 ± 1.33	17 ± 1.75	14 ± 1.03	19 ± 1.37

Numbers indicate mean diameter of growth inhibition zone (MDIZ)^∗^ in mm ± standard deviation. Interpretation of results: <9 mm zone was considered as inactive; 9–12 mm as partially active; 13–18 mm was active and >18 mm as very active, (-): No inhibition zone.

**Table 2:** Comparison of activity of citrus essential oils to a commercial 78% alcohol hand sanitizer as control using ANOVA followed by *post hoc* Tukey HSD.

	Grapefruit essential oil	Lime essential oil	Orange essential oil
Commercial 78% alcohol hand sanitizer (*p* < 0.01)	*E. coli*		
	ANOVA *F* = 51.067, *p*=0.001		
	*p*=0.001^*∗*^	*p*=0.001^*∗*^	*p*=0.001^*∗*^
	*P. aeruginosa*		
	ANOVA *F* = 8.082, *p*=0.001		
	*p*=0.064	*p*=0.285	*p*=0.001^*∗*^
	*S. aureus*		
	ANOVA *F* = 19.303, *p*=0.001		
	*p*=0.001^*∗*^	*p*=0.998	*p*=0 0.001^*∗*^
	*B. subtilis*		
	ANOVA *F* = 10.887, *p*=0.001		
	*p*=0.001^*∗*^	*p*=0.523	*p*=0.001^*∗*^
	*C. albicans*		
	ANOVA *F* = 18.219, *p*=0.001		
	*p*=0.001^*∗*^	*p*=0.371	*p*=0.001^*∗*^

^∗^The *p* value indicates significant difference between the essential oil and the commercial hand sanitizer as *p* < 0.01.

**Table 3:** Noninferiority test of citrus essential oils to a commercial 78% alcohol hand sanitizer as control.

	Grapefruit essential oil	Lime essential oil	Orange essential oil
Noninferiority test to a commercial 78% alcohol hand sanitizer (*p* < 0.01)	*E. coli*		
	*p*=0.999	*p*=0.999	*p*=0.998
	*P. aeruginosa*		
			*p*=0.990
	*S. aureus*		
	*p*=0.999		*p*=0.999
	*B. subtilis*		
	*p*=0.956		*p*=0.960
	*C. albicans*		
	*p*=0.997		*p*=0.999

**Table 4:** Comparison between antimicrobial activities of different types of citrus essential oils against *E. coli* using ANOVA followed by *post hoc* Tukey HSD.

Microorganism	Essential oil	Other essential oils	Mean dia. of inhibition zone (mm)	*p* value
ANOVA *F* = 11.649, *p*=0.001				

*E. coli*	Lime	Grapefruit	*M*_*L*_ = 12	*p*=0.032^*∗*^
			*M*_*G*_ = 10	
	Lime	Orange	*M*_*L*_ = 12	*p*=0.152
			*M*_*O*_ = 13	
	Grapefruit	Orange	*M*_*G*_ = 10	*p*=0.001^*∗*^
			*M*_*O*_ = 13	

^∗^The *p* value indicates significant difference between the essential oils as *p* < 0.05.

**Table 5:** Comparison between antimicrobial activities of different types of citrus essential oils against *P. aeruginosa* using ANOVA followed by *post hoc* Tukey HSD.

Microorganism	Essential oil	Other essential oils	Mean dia. of inhibition zone (mm)	*p* value
ANOVA *F* = 6.573, *p*=0.009				

*P. aeruginosa*	Lime	Grapefruit	*M*_*L*_ = 15	*p*=0 0.603
			*M*_*G*_ = 14	
	Lime	Orange	*M*_*L*_ = 15	*p*=0.008^*∗*^
			*M*_*O*_ = 12	
	Grapefruit	Orange	*M*_*G*_ = 14	*p*=0.056
			*M*_*O*_ = 12	

^∗^The *p* value indicates significant difference between the essential oils as *p* < 0.05.

**Table 6:** Comparison between antimicrobial activities of different types of citrus essential oils against *S. aureus* using ANOVA followed by *post hoc* Tukey HSD.

Microorganism	Essential oil	Other essential oils	Mean dia. of inhibition zone (mm)	*p* value
ANOVA *F* = 16.885, *p*=0 .001				

*S. aureus*	Lime	Grapefruit	*M*_*L*_ = 15	*p*=0.001^*∗*^
			*M*_*G*_ = 10	
	Lime	Orange	*M*_*L*_ = 15	*p*=0.001^*∗*^
			*M*_*O*_ = 10	
	Grapefruit	Orange	*M*_*G*_ = 10	*p*=0.987
			*M*_*O*_ = 10	

^∗^The *p* value indicates significant difference between the essential oils as *p* < 0.05.

**Table 7:** Comparison between antimicrobial activities of different types of citrus essential oils against *B. subtilis* using ANOVA followed by *post hoc* Tukey HSD.

Microorganism	Essential oil	Other essential oils	Mean dia. of inhibition zone (mm)	*p* value
ANOVA *F* = 10.208, *p*=0.002				

*B. subtilis*	Lime	Grapefruit	*M*_*L*_ = 16	*p*=0.004^*∗*^
			*M*_*G*_ = 13	
	Lime	Orange	*M*_*L*_ = 16	*p*=0.004^*∗*^
			*M*_*O*_ = 13	
	Grapefruit	Orange	*M*_*G*_ = 13	*p*=1.000
			*M*_*O*_ = 13	

^∗^The *p* value indicates significant difference between the essential oils as *p* < 0.05.

**Table 8:** Comparison between antimicrobial activities of different types of citrus essential oils against *C. albicans* using ANOVA followed by *post hoc* Tukey HSD.

Microorganism	Essential oil	Other essential oils	Mean dia. of inhibition zone (mm)	*p* value
ANOVA *F* = 12.062, *p*=0.001				

*C. albicans*	Lime	Grapefruit	*M*_*L*_ = 17	*p*=0.003^*∗*^
			*M*_*G*_ = 14	
	Lime	Orange	*M*_*L*_ = 17	*p*=0.001^*∗*^
			*M*_*O*_ = 14	
	Grapefruit	Orange	*M*_*G*_ = 14	*p*=0.813
			*M*_*O*_ = 14	

^∗^The *p* value indicates significant difference between the essential oils as *p* < 0.05.

**Table 9:** Chemical composition of grapefruit essential oil by GC-MS analysis.

Essential oil	Most abundant components	Formula	Chemical class	% area
Grapefruit				

1	D-Limonene	C_10_H_16_	Hydrocarbons	91.5
2	*β*-Myrcene	C_10_H_16_	Hydrocarbons	2.29
3	Trans-linalool oxide	C_10_H_18_O_2_	Oxide	1.54
4	Isocaryophyllene	C_15_H_24_	Hydrocarbons	1.20
5	6-Methyl-2-(2-oxiranyl)-5 hepten-2-ol	C_10_H_18_O_2_	Alcohols	0.79
6	*β*-Linalool	C_10_H_18_O	Alcohols	0.67
7	*α*-Pinene	C_10_H_16_	Hydrocarbons	0.65
8	*δ*-Cadinene	C_15_H_24_	Hydrocarbons	0.35
9	Copaene	C_15_H_24_	Hydrocarbons	0.32
10	*β*-Pinene	C_10_H_16_	Hydrocarbons	0.22
11	*β*-Copaene	C_15_H_24_	Hydrocarbons	0.17
12	Humulene	C_15_H_24_	Hydrocarbons	0.17
13	Germacrene	C_15_H_24_	Hydrocarbons	0.15
14	*γ*-Elemene	C_15_H_24_	Hydrocarbons	0.04
% Oxygenated compounds				3
% Hydrocarbon compounds				97
Monoterpenes C_10_H_16_ : sesquiterpenes C_15_H_24_				39.4

**Table 10:** Chemical composition of lime essential oil by GC-MS analysis.

Essential oil	Most abundant components	Formula	Chemical class	% area
Lime				

1	D-limonene	C_10_H_16_	Hydrocarbons	25.5
2	*γ*-Terpinene	C_10_H_16_	Hydrocarbons	11.3
3	*α*-Citral	C_10_H_16_O	Aldehydes	9.68
4	*β*-Pinene	C_10_H_16_	Hydrocarbons	9.39
5	Cis-citral	C_10_H_16_O	Aldehydes	8.09
6	Cis-geraniol	C_10_H_18_O	Alcohols	5.12
7	*α*-Terpineol	C_10_H_18_O	Alcohols	4.28
8	Geraniol	C_10_H_18_O	Alcohols	4.23
9	*β*-Bisabolene	C_15_H_24_	Hydrocarbons	4.05
10	Trans *α*-bergamotene	C_15_H_24_	Hydrocarbons	3.24
11	Isocaryophyllene	C_15_H_24_	Hydrocarbons	2.61
12	Terpinen-4-ol	C_10_H_18_O	Alcohols	2.18
13	*β*-Linalool	C_10_H_18_O	Alcohols	1.85
14	4(10)-Thujene	C_10_H_16_	Hydrocarbons	1.12
15	Cyclohexane, 1-ethenyl-1-methyl-2,4-bis(1-methylethenyl)-,[1S (1.alpha.,2.beta.,4.beta.)]-	C_15_H_24_	Hydrocarbons	1.02
16	Cyclohexene, 4-ethenyl-4-methyl-3-(1-methylethenyl)-1-(1-methylethyl)-,(3R-trans)-	C_15_H_24_	Hydrocarbons	1.00
17	2-Carene	C_10_H_16_	Hydrocarbons	0.89
18	*α*-Pinene	C_10_H_16_	Hydrocarbons	0.71
19	*β*-Myrcene	C_10_H_16_	Hydrocarbons	0.54
20	Germacrene D	C_15_H_24_	Hydrocarbons	0.42
21	*β*-Ocimene	C_10_H_16_	Hydrocarbons	0.40
22	Humulene	C_15_H_24_	Hydrocarbons	0.38
23	2(10) Pinen-3-ol, (1S, 3R, 5S)-(−)-	C_10_H_16_O	Alcohols	0.30
24	Endo-borneol	C_10_H_18_O	Alcohols	0.25
25	*γ*-Elemene	C_15_H_24_	Hydrocarbons	0.22
26	(+)-2-Carene	C_10_H_16_	Hydrocarbons	0.20
27	Benzene, 1-methyl-3-(1-methylethyl)-	C_10_H_14_	Hydrocarbons	0.18
28	3-Pinanone, cis	C_10_H_16_O	Ketone	0.18
29	*α*-Bergamotene	C_15_H_24_	Hydrocarbons	0.17
30	Cis-*α*-bisabolene	C_15_H_24_	Hydrocarbons	0.16
31	*α*-Phellandrene	C_10_H_16_	Hydrocarbons	0.09
32	2-Cyclohexen-1-ol,1-methyl-4-(1-methylethyl)	C_10_H_18_O	Alcohols	0.09
33	8-Decen-2-one, 9-methyl-5-methylene-	C_12_H_20_O	Ketone	0.09
34	Bicyclo[3.3.1]nonan-9-one,1,2,4-trimethyl-3-nitro-,(2-endo,3-exo,4-exo)-(.+-.)-	C_12_H_19_NO_3_	Nitro compounds	0.08
35	Camphene	C_10_H_16_	Hydrocarbons	0.04
% Oxygenated compounds				36.34
% Hydrocarbon compounds				63.6
Monoterpenes C_10_H_16_ : sesquiterpenes C_15_H_24_				3.7

**Table 11:** Chemical composition of orange essential oil by GC-MS analysis.

Essential oil	Most abundant components	Formula	Chemical class	% Area
Orange				

1	D-Limonene	C_10_H_16_	Hydrocarbons	95.39
2	*β*-Myrcene	C_10_H_16_	Hydrocarbons	3.02
3	*α*-Pinene	C_10_H_16_	Hydrocarbons	0.85
4	Cis-*β*-ocimene	C_10_H_16_	Hydrocarbons	0.44
5	4(10)-Thujene	C_10_H_16_	Hydrocarbons	0.18
6	*β*-Pinene	C_10_H_16_	Hydrocarbons	0.14
% Oxygenated compounds				0
% Hydrocarbon compounds				100
Monoterpenes C_10_H_16_ : Sesquiterpenes C_15_H_24_				100.0

## Data Availability

The data used to support the findings of this study are included within the article.
